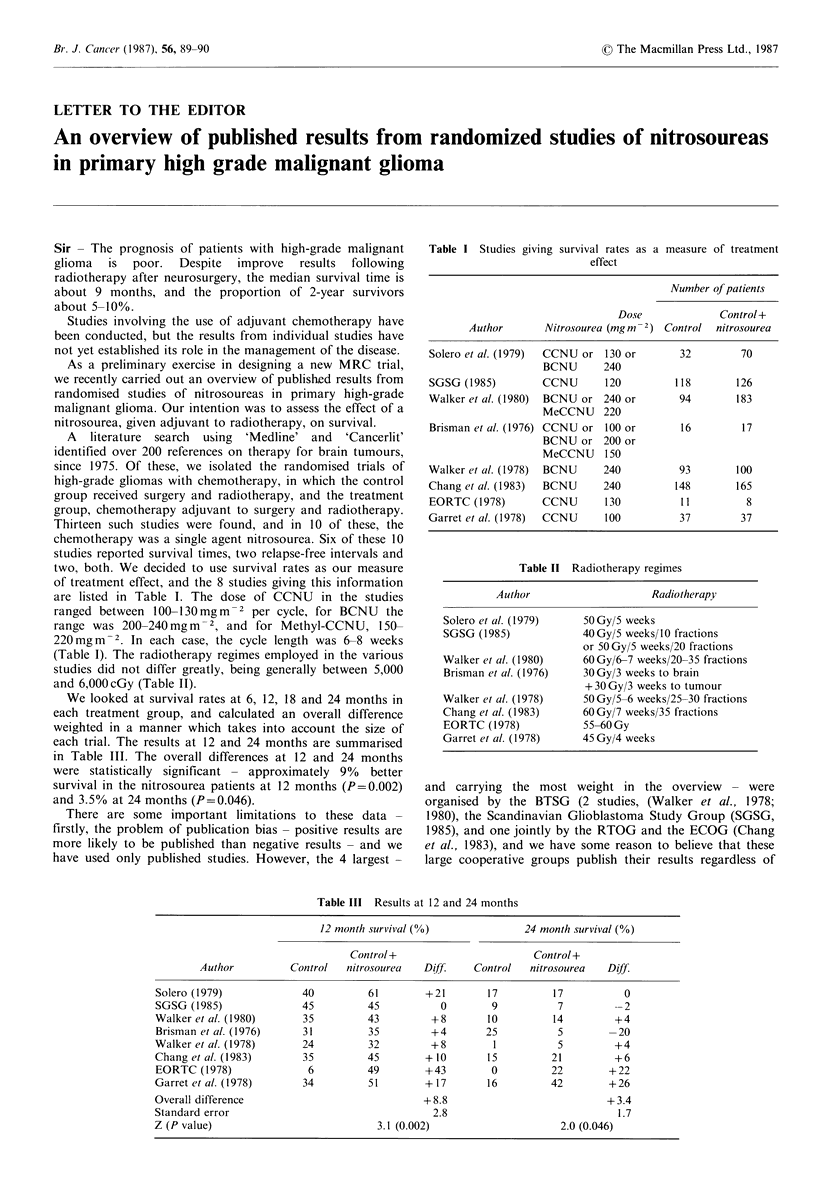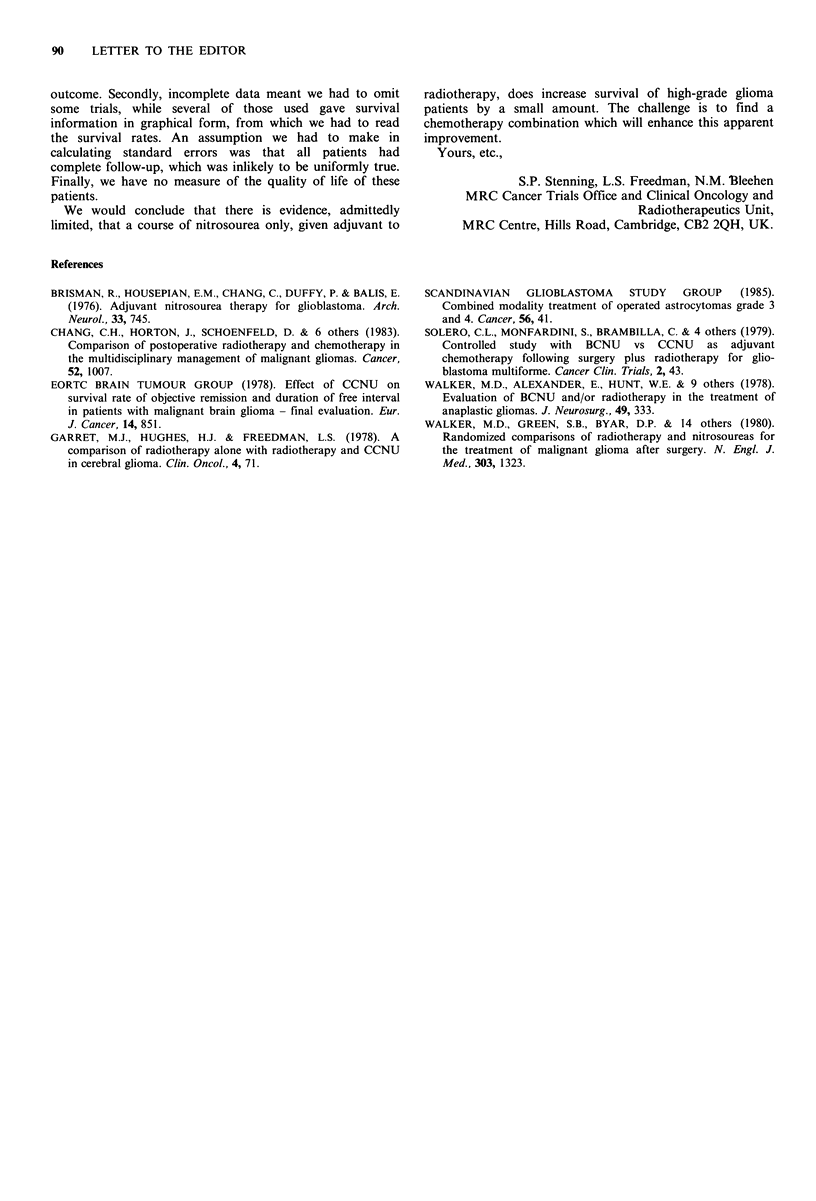# An overview of published results from randomized studies of nitrosoureas in primary high grade malignant glioma.

**DOI:** 10.1038/bjc.1987.161

**Published:** 1987-07

**Authors:** S. P. Stenning, L. S. Freedman, N. M. Bleehen


*Br. J. Cancer* (1987), **56**, 89–90 © The Macmillan Press Ltd., 1987

### LETTER TO THE EDITOR

### An overview of published results from randomized studies of nitrosoureas in primary high grade malignant glioma

**Sir** – The prognosis of patients with high-grade malignant glioma is poor. Despite improve results following radiotherapy after neurosurgery, the median survival time is about 9 months, and the proportion of 2-year survivors about 5–10%.

Studies involving the use of adjuvant chemotherapy have been conducted, but the results from individual studies have not yet established its role in the management of the disease.

As a preliminary exercise in designing a new MRC trial, we recently carried out an overview of published results from randomised studies of nitrosoureas in primary high-grade malignant glioma. Our intention was to assess the effect of a nitrosourea, given adjuvant to radiotherapy, on survival.

A literature search using 'Medline' and 'Cancerlit' identified over 200 references on therapy for brain tumours, since 1975. Of these, we isolated the randomised trials of high-grade gliomas with chemotherapy, in which the control group received surgery and radiotherapy, and the treatment group, chemotherapy adjuvant to surgery and radiotherapy. Thirteen such studies were found, and in 10 of these, the chemotherapy was a single agent nitrosourea. Six of these 10 studies reported survival times, two relapse-free intervals and two, both. We decided to use survival rates as our measure of treatment effect, and the 8 studies giving this information are listed in Table I. The dose of CCNU in the studies ranged between 100–130 $mg\,m^{-2}$ per cycle, for BCNU the range was 200–240 $mg\,m^{-2}$, and for Methyl-CCNU, 150–220 $mg\,m^{-2}$. In each case, the cycle length was 6–8 weeks (Table I). The radiotherapy regimes employed in the various studies did not differ greatly, being generally between 5,000 and 6,000 cGy (Table II).

We looked at survival rates at 6, 12, 18 and 24 months in each treatment group, and calculated an overall difference weighted in a manner which takes into account the size of each trial. The results at 12 and 24 months are summarised in Table III. The overall differences at 12 and 24 months were statistically significant – approximately 9% better survival in the nitrosourea patients at 12 months ($P=0.002$) and 3.5% at 24 months ($P=0.046$).

There are some important limitations to these data – firstly, the problem of publication bias – positive results are more likely to be published than negative results – and we have used only published studies. However, the 4 largest – and carrying the most weight in the overview – were organised by the BTSG (2 studies, (Walker *et al.*, 1978; 1980), the Scandinavian Glioblastoma Study Group (SGSG, 1985), and one jointly by the RTOG and the ECOG (Chang *et al.*, 1983), and we have some reason to believe that these large cooperative groups publish their results regardless of

**Table I** Studies giving survival rates as a measure of treatment effect

| | | | Number of patients | |
|---|---|---|---|---|
| *Author* | *Nitrosourea* | *Dose* ($mg\,m^{-2}$) | *Control* | *Control + nitrosourea* |
| Solero *et al.* (1979) | CCNU or BCNU | 130 or 240 | 32 | 70 |
| SGSG (1985) | CCNU | 120 | 118 | 126 |
| Walker *et al.* (1980) | BCNU or MeCCNU | 240 or 220 | 94 | 183 |
| Brisman *et al.* (1976) | CCNU or BCNU or MeCCNU | 100 or 200 or 150 | 16 | 17 |
| Walker *et al.* (1978) | BCNU | 240 | 93 | 100 |
| Chang *et al.* (1983) | BCNU | 240 | 148 | 165 |
| EORTC (1978) | CCNU | 130 | 11 | 8 |
| Garret *et al.* (1978) | CCNU | 100 | 37 | 37 |

**Table II** Radiotherapy regimes

| *Author* | *Radiotherapy* |
|---|---|
| Solero *et al.* (1979) | 50 Gy/5 weeks |
| SGSG (1985) | 40 Gy/5 weeks/10 fractions or 50 Gy/5 weeks/20 fractions |
| Walker *et al.* (1980) | 60 Gy/6–7 weeks/20–35 fractions |
| Brisman *et al.* (1976) | 30 Gy/3 weeks to brain + 30 Gy/3 weeks to tumour |
| Walker *et al.* (1978) | 50 Gy/5–6 weeks/25–30 fractions |
| Chang *et al.* (1983) | 60 Gy/7 weeks/35 fractions |
| EORTC (1978) | 55–60 Gy |
| Garret *et al.* (1978) | 45 Gy/4 weeks |

**Table III** Results at 12 and 24 months

| | 12 month survival (%) | | | 24 month survival (%) | | |
|---|---|---|---|---|---|---|
| *Author* | *Control* | *Control + nitrosourea* | *Diff.* | *Control* | *Control + nitrosourea* | *Diff.* |
| Solero (1979) | 40 | 61 | +21 | 17 | 17 | 0 |
| SGSG (1985) | 45 | 45 | 0 | 9 | 7 | −2 |
| Walker *et al.* (1980) | 35 | 43 | +8 | 10 | 14 | +4 |
| Brisman *et al.* (1976) | 31 | 35 | +4 | 25 | 5 | −20 |
| Walker *et al.* (1978) | 24 | 32 | +8 | 1 | 5 | +4 |
| Chang *et al.* (1983) | 35 | 45 | +10 | 15 | 21 | +6 |
| EORTC (1978) | 6 | 49 | +43 | 0 | 22 | +22 |
| Garret *et al.* (1978) | 34 | 51 | +17 | 16 | 42 | +26 |
| Overall difference | | | +8.8 | | | +3.4 |
| Standard error | | | 2.8 | | | 1.7 |
| Z (*P* value) | | 3.1 (0.002) | | | 2.0 (0.046) | |

outcome. Secondly, incomplete data meant we had to omit some trials, while several of those used gave survival information in graphical form, from which we had to read the survival rates. An assumption we had to make in calculating standard errors was that all patients had complete follow-up, which was inlikely to be uniformly true. Finally, we have no measure of the quality of life of these patients.

We would conclude that there is evidence, admittedly limited, that a course of nitrosourea only, given adjuvant to radiotherapy, does increase survival of high-grade glioma patients by a small amount. The challenge is to find a chemotherapy combination which will enhance this apparent improvement.

Yours, etc.,

S.P. Stenning, L.S. Freedman, N.M. Bleehen
MRC Cancer Trials Office and Clinical Oncology and Radiotherapeutics Unit,
MRC Centre, Hills Road, Cambridge, CB2 2QH, UK.

**References**

BRISMAN, R., HOUSEPIAN, E.M., CHANG, C., DUFFY, P. & BALIS, E. (1976). Adjuvant nitrosourea therapy for glioblastoma. *Arch. Neurol.*, **33**, 745.

CHANG, C.H., HORTON, J., SCHOENFELD, D. & 6 others (1983). Comparison of postoperative radiotherapy and chemotherapy in the multidisciplinary management of malignant gliomas. *Cancer*, **52**, 1007.

EORTC BRAIN TUMOUR GROUP (1978). Effect of CCNU on survival rate of objective remission and duration of free interval in patients with malignant brain glioma – final evaluation. *Eur. J. Cancer*, **14**, 851.

GARRET, M.J., HUGHES, H.J. & FREEDMAN, L.S. (1978). A comparison of radiotherapy alone with radiotherapy and CCNU in cerebral glioma. *Clin. Oncol.*, **4**, 71.

SCANDINAVIAN GLIOBLASTOMA STUDY GROUP (1985). Combined modality treatment of operated astrocytomas grade 3 and 4. *Cancer*, **56**, 41.

SOLERO, C.L., MONFARDINI, S., BRAMBILLA, C. & 4 others (1979). Controlled study with BCNU vs CCNU as adjuvant chemotherapy following surgery plus radiotherapy for glioblastoma multiforme. *Cancer Clin. Trials*, **2**, 43.

WALKER, M.D., ALEXANDER, E., HUNT, W.E. & 9 others (1978). Evaluation of BCNU and/or radiotherapy in the treatment of anaplastic gliomas. *J. Neurosurg.*, **49**, 333.

WALKER, M.D., GREEN, S.B., BYAR, D.P. & 14 others (1980). Randomized comparisons of radiotherapy and nitrosoureas for the treatment of malignant glioma after surgery. *N. Engl. J. Med.*, **303**, 1323.